# The enemy as animal: Symmetric dehumanization during asymmetric warfare

**DOI:** 10.1371/journal.pone.0181422

**Published:** 2017-07-26

**Authors:** Emile Bruneau, Nour Kteily

**Affiliations:** 1 Annenberg School for Communication, University of Pennsylvania, Philadelphia, PA, United States of America; 2 Department of Brain and Cognitive Sciences, Massachusetts Institute of Technology, Cambridge, MA, United States of America; 3 Kellogg School of Management, Northwestern University, Chicago, IL, United States of America; University of Reading, UNITED KINGDOM

## Abstract

Historically, dehumanization has enabled members of advantaged groups to ‘morally disengage’ from disadvantaged group suffering, thereby facilitating acts of intergroup aggression such as colonization, slavery and genocide. But is blatant dehumanization exclusive to those at the top ‘looking down’, or might disadvantaged groups similarly dehumanize those who dominate them? We examined this question in the context of intergroup warfare in which the disadvantaged group shoulders a disproportionate share of casualties and may be especially likely to question the humanity of the advantaged group. Specifically, we assessed blatant dehumanization in the context of stark asymmetric conflict between Israelis (Study 1; *N* = 521) and Palestinians (Study 2; *N* = 354) during the 2014 Gaza war. We observed that (a) community samples of Israelis and Palestinians expressed extreme (and comparable) levels of blatant dehumanization, (b) blatant dehumanization was uniquely associated with outcomes related to outgroup hostility for both groups, even after accounting for political ideologies known to strongly predict outgroup aggression, and (c) the strength of association between blatant dehumanization and outcomes was similar across both groups. This study illuminates the striking potency and symmetry of blatant dehumanization among those on both sides of an active asymmetric conflict.

## Introduction

“Wars begin in the minds of men.”–United Nations Educational, Scientific and Cultural Organization (UNESCO) constitution preamble

Humans have in place strong moral prohibitions and psychological restraints against harming others. At the same time, history illustrates that humans have a remarkable propensity to commit extreme violence, particularly across group boundaries [1]. Writing in the shadow of World War II and the Holocaust, many psychologists suggested that the horrors committed by the Nazis against Jews, the Roma and others was enabled by the perception of these groups as ‘sub-human’, which led to ‘moral disengagement’ from their suffering (e.g., [2], [3]). And long before the Nazi regime, dehumanization was regularly employed by European powers as they colonized, enslaved and exterminated other groups. For example, during the colonization of Ireland by the English starting in the 17^th^ Century, the Irish were regularly depicted in newspapers as culturally inferior, irrational and prone to violence, a dehumanizing stereotype that was popularized through the ‘Irish Joke’ [4]. Similarly, Aborigines, Native Americans and Africans were depicted as infantile savages, to be variously disposed of, enslaved or adopted as the “White man’s burden” [5].

The contexts of genocide, colonization and slavery provide us with examples of dehumanization applied down the power gradient, by those in power against the conquered masses. But is dehumanization a psychological weapon exclusively wielded by the ‘oppressor’? As proof of principle, we sought here to identify a context in which overt dehumanization might be demonstrated to occur bi-directionally up and down the power gradient: in the midst of an active (i.e., ‘hot’) and violent asymmetric conflict.

Although empirical work over the past two decades has focused primarily on subtle forms of dehumanization (e.g., infrahumanization; [6]), recent work has shifted to (re-)examining more blatant incarnations [7], [8], [9]. For example, [10] developed a measure based on the popular ‘Ascent of Man’ diagram, which was specifically designed to capture blatant dehumanization by assessing how ‘evolved and civilized’ people considered other groups to be. This work showed that blatant dehumanization is strongly and uniquely associated with the types of highly aggressive attitudes that are particularly relevant to violent conflict, such as support for torture and militaristic forms of counter-terrorism. However, the research focused exclusively on the perceptions of advantaged groups (Americans, British people, Hungarians) with respect to disadvantaged groups (e.g., Arabs, Muslims, the Roma). Here, we sought to apply this measure to both sides of an active conflict: Israelis and Palestinians during the 2014 War in Gaza.

By examining the level and potency of blatant dehumanization down the power gradient during active conflict (from empowered Israelis towards disempowered Palestinians), we examine dehumanization during one of the contexts which originally inspired theoretical work on dehumanization. However, the primary goal of this research was to examine blatant dehumanization in the opposite direction: from disempowered Palestinians towards empowered Israelis. Although much recent research suggests that dehumanization down the power gradient is likely to be strong and potent in this context, there are arguments supporting competing predictions about dehumanization in the opposite direction.

On the one hand, hierarchical models of intergroup relations provide evidence that social hierarchies are often endorsed by both disadvantaged and advantaged groups. For example, there is often a surprising level of consensus among members of advantaged and disadvantaged groups in their endorsement of ‘hierarchy enhancing legitimizing myths’, such as the belief in meritocracy or the protestant work ethic [11]. Other work further suggests that individuals, including those belonging to disadvantaged groups, in fact have a motivation to justify the social system because of a desire for order and predictability [12]. Consistent with this, disadvantaged groups sometimes display implicit bias against their own group [13], [14]. It is also the case that high-status individuals and groups tend to be stereotyped as highly competent, possessing traits such as being capable, intelligent, and sophisticated (e.g., [15]) that are associated with full humanness (e.g., [16]). From these perspectives, one might predict that disadvantaged groups like Palestinians might show a lower tendency to dehumanize an advantaged outgroup like Israelis than the reverse, or even that they might rate the outgroup as equally human or *more* human than the ingroup.

However, the degree to which disadvantaged or disempowered groups favor advantaged groups and legitimize a system of oppression is likely to be limited by a range of factors—we suggest that one of those factors could be the perceived brutality of the other group. Although factors such as the massive disparity in economic development, military might and scientific achievement between Israel and Palestine might lead some Palestinians to support the view that Israelis are more ‘advanced’ or ‘sophisticated’ than Palestinians, the history of violent conflict between the two groups should also lead Palestinians to perceive Israelis as highly ‘savage’, ‘aggressive’ and ‘cold-hearted’—perceptions that are strongly associated with Ascent dehumanization ([10], Study 5). As the 2014 Gaza war unfolded and ingroup casualties mounted, such perceptions are likely to have been particularly salient, making this an ideal context in which blatant dehumanization up the power gradient might emerge (see also [17]). This view is also in line with social identity approaches to intergroup relations, which suggest that individuals generally favor their own groups over outgroups across a range of evaluations and behaviors, a pattern that can escalate to outgroup derogation under conditions of conflict [18]. From this perspective, disadvantaged groups may be predicted to dehumanize advantaged groups, particularly those with whom intergroup conflict is salient.

In the current research, we predicted that the active conflictual relationship between Israelis and Palestinians would overcome any tendency to humanize the advantaged outgroup, resulting in high levels of blatant dehumanization among disempowered Palestinians towards empowered Israelis, potentially comparable to those we expected among Israelis towards Palestinians. We further expected that blatant dehumanization by the disempowered group towards the empowered group would be uniquely predictive of conflict-relevant outcomes, just as blatant dehumanization by empowered groups predict such outcomes. Confirmation of these predictions would serve as a strong proof-of-principle that blatant dehumanization can occur and function similarly both up and down a power gradient during asymmetric conflict.

We examined these questions by exploring the prevalence and consequences of blatant dehumanization within community samples of Israelis and Palestinians that were broadly comparable in terms of gender, education and conservatism. In these populations, we assess both mean levels of blatant outgroup dehumanization, as well as the unique relationship between blatant dehumanization and support for conflict relevant behaviors, beyond ideological variables (i.e., conservatism and social dominance orientation) known to strongly drive these attitudes [19], [20], [21], [22].

Although the focus of this research is on dehumanization up the power gradient, we also extend prior work by engaging in a comparative analysis that directly examines the role of group status and power on blatant dehumanization. Previous work suggests that feelings of power are associated with subtle dehumanization [23], however the effect of actual or perceived power on blatant dehumanization has not been examined. In the current study we investigate the role of group power on blatant dehumanization in two related ways. First, we examine the role of group position: Wherever we have the same outcome measures among both groups, we determine whether the predictive utility of blatant dehumanization is statistically moderated by membership in the advantaged versus disadvantaged group. Second, beyond group membership, we examine the role of individual-level variation in the subjective sense of group power that individuals report. Specifically, among our samples of Israelis and Palestinians, we determine whether *feeling* more or less powerful influences (a) levels of blatant outgroup dehumanization and (b) the association between blatant dehumanization and outcome measures. We investigate these important questions in a unique and important context (the 2014 Gaza war), when dehumanization and aggression were highly relevant.

## Study 1

### Method

Research was approved by the Institutional Review Board (IRB) at the Massachusetts Institute of Technology.

#### Participants

We aimed to collect data from as large a sample as was feasible. The first author collected data online from 521 Israeli participants in early August 2014 (i.e., during the War in Gaza; *M* age = 39.65; *SD* = 12.74; 51.2% male), using the Midgam panel service (see also [17]). The survey was open to participants from any region of Israel. The political orientation of the sample was heterogeneous, though it leaned somewhat conservative with 57.6% of respondents reporting that they were slightly, strongly or extremely right (see Table 1 for descriptive details). The sample was generally well educated, with 67.3% completing college or professional school. Participants completed an omnibus survey (in Hebrew) of their political and social attitudes. We discuss below the variables relating centrally to the current research on blatant dehumanization (see supplementary materials for complete survey).

**Table 1:** Descriptive statistics and variable intercorrelations for Study 1.

	1	2	3	4	5	6	7	8	9	10	11
1. Perceived Group Power	-										
2. Social Dominance Orientation	.11*	-									
3. Conservatism	.01	.37***	-								
4. Blatant Dehumanization	.14**	.33***	.35***	-							
5. Hope	.05	-.36***	-.55***	-.34***	-						
6. Hostile Emotions	.05	.33***	.50***	.47***	-.45***	-					
7. Group-Based Guilt	-.07	-.28***	-.51***	-.29***	.35***	-.34***	-				
8. Willingness to Negotiate	-.02	-.26***	-.55***	-.33***	.52***	-.35***	.36***	-			
9. Concession Making	-.04	-.31***	-.61***	-.41***	.56***	-.47***	.52***	.60***	-		
10. Collective Aggression	.03	.44***	.58***	.46***	-.54***	.56***	-.48***	-.50***	-.59***	-	
11. Acceptance of Civilian Casualties	.02	.27***	.34***	.26***	-.32***	.36***	-.35***	-.31***	-.40***	.51***	-
*M*	83.99	40.74	62.67	39.81	46.89	57.54	16.44	40.90	31.89	55.82	575.08
*SD*	24.27	18.59	20.18	35.01	28.16	23.39	23.76	27.70	25.96	21.00	456.49
Quartiles	66.67, 100, 100	28.57, 40.48, 52.38	50.00, 66.67, 83.33	5.00, 40.00, 69.00	25.00, 50.00, 70.83	38.89, 55.55, 72.22	0.00, 0.00, 25.00	16.67, 41.67, 58.33	8.33, 29.17, 50.00	41.67, 54.17, 70.31	26.00, 990.00, 1000.00

*** p < .001

** p < .01

* p < .05

#### Measures

Unless otherwise noted, all measures were converted from the scales reported below to a 0–100 scale, for ease of comparison with the results of Study 2.

#### Age and gender

Participants reported their age and gender (1 = male; 2 = female).

#### Education

Participants indicated their education by selecting one of 14 options, which were broken into five major categories reflecting whether participants had: 1 = Completed primary education; 2 = Begun or completed secondary education; 3 = Completed some college or professional higher education; 4 = Completed College or professional school; 5 = Begun or completed graduate school.

#### Social dominance orientation

SDO was assessed using 8 items from the dominance sub-dimension of the SDO-7 scale [24], which relates most strongly to the types of aggressive attitudes we were interested in exploring in this work. Responses were given on Likert scales anchored at 1 (‘Strongly Disagree’) and 7 (‘Strongly Agree’). Removing one item (“An ideal society requires some groups to be on top and others to be on the bottom”) improved scale reliability; the remaining seven items formed a reliable scale (α = .73).

#### Conservatism

Participants indicated their political ideology on a 7-point scale, anchored at 1 (‘Extreme right’) and 7 (‘Extreme left’); we reverse-scored participants’ responses such that higher scores indicate more conservative attitudes.

#### Perceived group power

We assessed subjective perceptions of group power by asking participants to respond to the following item: “In general, when you think about power relations between Israel and the Palestinians, which side do you think has more power?” Responses were provided on a 1 (‘Definitely Palestinians’) to 7 (‘Definitely Israel’) scale. We also assessed perceptions of power on a broader array of specific dimensions (e.g., with respect to military power, economic power, and public sympathy). We focused our assessment on the item noted above because it intentionally captures a global assessment of group power, but results using a composite of the specific items mentioned here provided similar conclusions.

#### Blatant dehumanization

We assessed blatant dehumanization of Palestinians using the Ascent scale of dehumanization [10]. Participants were shown the ‘Ascent of Man’ image, and asked to rate on this scale how ‘evolved and civilized’ they perceived the average member of each of the following groups to be: Europeans, Palestinians, Arabs, Muslims, Israelis, Residents of Gaza, Members of Hamas, Americans, and Arab-Israelis. Responses were provided on a scale marked only at its anchors of 0 (‘left side of the above image’) and 100 (‘right side of the above image’). The left side of the image corresponded to a quadrupedal ancestor of modern humans, and the right side of the image to a modern human. As in previous work [10], we computed dehumanization scores by subtracting Palestinian (i.e., outgroup) humanity ratings from Israeli (i.e., ingroup) humanity ratings, such that higher scores indicate greater outgroup dehumanization (conclusions were similar when using absolute outgroup ratings). Our central interest in this work was examining blatant dehumanization, and thus, we focus on this construct. We also had a measure of secondary emotion attributions towards Palestinians. Controlling for this variable produced broadly similar results. For mean dehumanization responses across all target groups, see S1 Table.

#### Emotional hostility

Previous research has shown that blatant dehumanization is associated with hostile emotions towards the outgroup [10]. We assessed emotional hostility by asking participants to rate the extent to which they felt the following emotions towards Palestinians: hatred, hostility, and anger (α = .70). Responses were made on Likert scales anchored at 1 (‘Not at all’) and 7 (‘Very much so’).

#### Group-based guilt

We assessed group-based guilt by asking participants to rate the extent to which they felt “guilt regarding Israel’s treatment of the Palestinians”, and “Shame regarding Israel’s behavior towards the Palestinians”. We reasoned that individuals who dehumanized Palestinians would be less likely to feel guilty about the ingroup’s actions towards them, consistent with theorizing on moral disengagement as a consequence of dehumanization [2]. Responses were made on Likert scale anchored at 1 (‘Not at all’) and 7 (‘Very much so’) (*r* = .77, *p* < .001).

#### Hope

We assessed hope as a potential outcome of dehumanization, given recent research describing its central role in conflict resolution [25]. We reasoned that individuals who dehumanized the outgroup may be more likely to see negative outgroup attributes as fixed and thus be less likely to be hopeful about the likelihood of achieving peace. Hope was assessed with respect to the Israeli-Palestinian conflict using four items adapted from [25]. Sample item: “I have hope regarding the peaceful resolution of the Israeli-Palestinian conflict”. Participants indicated their responses (α = .84) on Likert scales anchored at 1 (‘Strongly Disagree’) and 7 (‘Strongly Agree’).

#### Willingness to negotiate

We reasoned that individuals who dehumanized another group would be more likely to take extreme positions and less open to engage the outgroup in the give-and-take often required of negotiations. We assessed willingness to negotiate with Palestinians using two items adapted from [26]: “How willing would you be for Israel to negotiate with a Palestinian side led by Mahmoud Abbas?” and “How willing would you be for Israel to enter direct negotiations involving Hamas in order to reach a final settlement?” Participants provided responses on Likert scales anchored at 1 (‘Not at all’) and 7 (‘Very much so’) (*r* = .47, *p* < .001).

#### Concession making

Previous research suggests that dehumanization may be associated with a lack of willingness to make concessions [27]. We examined willingness to make concessions in order to reach a final resolution with the Palestinians using a four-item scale adapted from [28]. These items included ratings of support for joint sovereignty over the holy sites in Jerusalem, and renouncement of Israeli control of Gaza, the West Bank, and Arab neighborhoods in East Jerusalem (α = .76). Responses were made on Likert scales anchored at 1 (‘Not at all’) and 7 (‘Very much so’).

#### Collective aggression

Individuals who blatantly dehumanize another group are more willing to engage in collective aggression towards that group [10]. We examined support for collective aggression using eight items. Sample items: “As long as Hamas continues to fire rockets at Israel, I think it is justified to bomb Palestinian schools and hospitals”, “I support hurting Palestinians in order to ‘teach them a lesson’” (α = .78). Participants responded on Likert scales anchored at 1 (‘Strongly Disagree’) and 7 (‘Strongly Agree’).

#### Acceptance of civilian casualties

During wartime, blatant dehumanization of outgroup is hypothesized to reduce concern over the welfare of outgroup civilians [29]. To assess acceptance of civilian casualties, we had participants respond to the following question: “Assume the following scenario is taking place: An Israeli soldier is fighting with his combat unit in a neighborhood in Gaza. He is shot and wounded by a Palestinian militant. In order to save him, one of the other soldiers in the combat unit needs to fire a mortar shell back. However, this is a dense urban neighborhood that contains Palestinian civilians. What is the maximum number of Palestinian civilian casualties that you think is acceptable in order to save the soldier’s life?” Participants were asked to choose the number of Palestinians casualties beyond which they would no longer endorse the bombing, and were restricted to a number between 0 and 1,000.

### Results

Descriptive statistics and variable inter-correlations can be found in Table 1. As expected, Israelis perceived their own group as significantly higher in power relative to the Palestinians (*M* = 83.99, *SD* = 24.27; one-sample t-test relative to the scale midpoint of 50: *t*(508) = 31.60, *p* < .001; see Table 1).

Our central interest was in examining blatant dehumanization. On average, Israelis rated Palestinians 39.81 points lower than their own group on the 0–100 Ascent scale. Strong blatant dehumanization was clearly a normative response in this context: a full 50% of the sample indicated a relative dehumanization score above 40, and 25% of the sample rated Palestinians over 69 points lower than their own group (see Table 1). Notably, by absolute rating Israelis regarded Palestinians (*M* = 41.21, *SD* = 32.46) as significantly closer to the quadrupedal human ancestor than the ‘fully evolved’ modern human (i.e., 100) on the Ascent diagram, *t*(510) = 6.13, *p* < .001.

Israelis’ hostile orientation towards Palestinians was also expressed across the outcome measures: participants in our sample reported experiencing strong negative emotions towards Palestinians, exhibited relatively low willingness to negotiate, low support for concession making, high support for collective aggression, and strikingly high acceptance of civilian casualties. On average, participants indicated that they would be willing to kill 575 Palestinian civilians in order to save the life of one Israeli soldier wounded by a Palestinian militant. The median for this measure in our sample was 990 Palestinians, and the modal response was the maximum value allowed (i.e., 1000 Palestinians, selected by 49.9% of the sample).

We next examined the extent to which blatant dehumanization was associated with each of these outcome measures, controlling for perceived group power, political ideology (SDO and conservatism), and demographics (age, gender, and education) (see Table 2). In line with prior work [30], [31] we found that conservatism significantly predicted all of the hostile outcome measures, and SDO predicted 4 of the 7 outcomes (less hope, more emotional hostility, greater collective aggression, and accepting a higher number of Palestinian civilian casualties). Despite the strong predictive power of SDO and conservatism, blatant dehumanization was uniquely associated with all of the outcome variables after controlling for these ideological variables, subjective perceptions of power, and demographic variables. Excluding demographics did not affect results here (or in Study 2), and results for all outcome measures were significant or marginally significant controlling for attribution of secondary emotions to Palestinians, with the exception of group-based guilt.

**Table 2:** Simultaneous regressions predicting outgroup attitudes as a function of political ideology and dehumanization in Study 1.

	Hope R^2^ = .36		Hostile Emotions R^2^ = .36		Group-Based Guilt R^2^ = .29			Willingness to Negotiate R^2^ = .34		Concession Making R^2^ = .42		Collective Aggression R^2^ = .45		Acceptance of Civilian Casualties R^2^ = .15	
	β	95% CI	β	95% CI	β	95% CI		β	95% CI	β	95% CI	β	95% CI	β	95% CI
Perceived Group Power	.10*	.02, .17	-.01	-.08, .07	-.05		-.13, .02	.01	-.06, .09	.00	-.07, .07	-.04	-.10, .03	-.01	-.10, .07
SDO	-.15***	-.23, -.07	.10*	.02, .18	-.07†	-.16, .01		-.03	-.11, .05	-.04	-.12, .03	.20***	.13, .27	.14**	.05, .23
Conservatism	-.45***	-.53 -.37	.35***	.27, .43	-.45***	-.53, -.36		-.46***	-.54, -.38	-.52***	-.60, -.45	.40***	.33, .48	.24***	.15, .34
Age	-.04	-.12, .03	-.02	-.09, .06	.01	-.07, .08		.14***	.06, .21	.01	-.06, .08	-.06	-.12, .01	.03	-.06, .12
Gender	-.03	-.10, .04	.07	-.00, .14	-.06	-.13, .02		.02	-.06, .09	-.02	-.08, .05	-.03	-.10, .04	.02	-.06, .11
Education	.03	-.05, .10	-.02	-.09, .06	-.04	-.11, .04		.02	-.06, .09	.05	-.03, .12	-.03	-.09, .04	-.05	-.13, .04
Blatant Dehumanization	-.14***	-.22, -.06	.31***	.23, .39	-.10*	-.18, -.02		-.14**	-.22, -.06	-.20***	-.28, -.13	.24***	.17, .32	.12*	.03, .21

*** *p* < .001

** *p* < .01

* *p* < .05

† *p* < .10

Finally, we examined how variation in the level of Israelis’ subjective perceptions of the power dynamics between their group and the Palestinians was associated with each of (a) blatant dehumanization of Palestinians, and (b) the predictive utility of dehumanization. We observed a weak positive correlation (see Table 1) such that Israelis who felt that their group was more powerful were significantly more likely to blatantly dehumanize Palestinians. Using the PROCESS macro ([32]; Model 1), we then examined whether subjective group power moderated the association between blatant dehumanization and the various outcome measures (controlling for all covariates). We observed little evidence consistent with this idea: although the negative association between blatant dehumanization and group-based guilt was stronger among Israelis who felt more powerful (*b* = -.07, *p* = .04, 95% CI [-.15, -.00]), we observed no significant interactions between subjective power and blatant dehumanization on hope (*b* = -.04, *p* = .31, 95% CI [-.11, .03]), emotional hostility (*b* = .03, *p* = .34, 95% CI [-.04, .10]), willingness to negotiate (*b* = -.01, *p* = .72, 95% CI [-.08, .06], concession making (*b* = -.01, *p* = .84, 95% CI [-.07, .06], collective aggression (*b* = .04, *p* = .20, 95% CI [-.02, .11], or collateral damage (*b* = -.00, *p* = .99, 95% CI [-.08, .08].

### Discussion

During the 2014 Gaza war, our sample of Israelis (members of the high power group) expressed extremely hostile attitudes towards Palestinians (members of the low power group), including the highest levels of blatant dehumanization towards any outgroup observed to date using the ‘Ascent of man’ measure of blatant dehumanization (i.e., higher than has been observed among American, English and Hungarian participants rating over two dozen different groups, including ISIS; [10], [33], [34]). Indeed, we found that Israelis rated Palestinians closer to an animal on the scale than to a ‘fully evolved’ human. This blatant dehumanization of Palestinians was also potent: even beyond subjective ratings of group power and ideological variables known to be strongly associated with intergroup hostility in this context (i.e., SDO and conservatism), dehumanization was uniquely associated with hostile attitudes likely to perpetuate cycles of intergroup conflict [29], [33], including unwillingness to negotiate, collective aggression and endorsement of massive civilian casualties. Interestingly, although we observed that the subjective perception of group power was weakly correlated with higher levels of blatant dehumanization, the association between dehumanization and outcomes was equally strong across levels of perceived power.

Although we expected that advantaged group members would blatantly dehumanize the ‘enemy’ during wartime, the *degree* of dehumanization, the vehemence of behavioral endorsements (particularly the number of Palestinian civilians that Israelis were willing to kill in order to save one Israeli soldier), and the association between dehumanization and outcomes beyond ideological variables were striking.

We next considered our central question of interest: whether disadvantaged Palestinians would similarly express blatant dehumanization towards Israelis in this same context, and if so, whether this dehumanization would similarly predict intergroup hostility.

## Study 2

According to the World Bank [35], Israel is a ‘high income developed country’, with one of the world’s most advanced militaries. By contrast, with a GDP only 3% of Israel’s and no standing army, Palestine is on par with ‘developing countries’ in the Middle East and North Africa. This objective power asymmetry between Israelis and Palestinians is reflected by the subjective perceptions of both Israelis and Palestinians, who were both found in prior work to perceive that Israel is the more powerful group in the conflict [26].

Given prior research on system justification [12] and work suggesting consensual support for hierarchy-legitimizing ideologies [11], it is possible that Palestinians might come to justify elements of their disadvantaged position, including by being less likely to dehumanize their more ‘developed’ and ‘sophisticated’ neighbor (see also [36], [37]). However, the amount of destruction and civilian death experienced by the Palestinian community during the war in Gaza prompted many Palestinians to focus on (what they perceived as) Israeli ‘brutality’ and ‘savagery’, animalistic traits associated with blatant dehumanization ([10], Study 5). Moreover, research suggests that conflict and associated feelings of illegitimacy may decrease processes like system justification [12] and increase the potential for outgroup derogation [18] and dehumanization [38]. For these reasons, we predicted that despite their disadvantaged position, Palestinians would express high levels of blatant dehumanization towards Israelis, providing an important proof-of-principle for the idea that blatant dehumanization can prevail up (and not just down) the power gradient.

### Method

Research was approved by the Institutional Review Board (IRB) at Northwestern University.

#### Participants

As with Study 1, we aimed to collect as large a sample as was feasible in Study 2: We thus collected data from 354 Palestinian participants online in late August 2014 (i.e., during the Gaza war; *M* age = 27.11; *SD* = 8.90; 55.8% male). Participants were community residents of the West Bank, recruited through a text-based employment agency (‘Souktel’). Participants received a text message via cell phone and were given the opportunity to participate in the study online in return for phone credit. As with the Israeli sample, the Palestinian sample was politically heterogeneous (see Table 3 for descriptive details), though it again leaned somewhat conservative, with 61.7% reporting at least moderate levels of conservatism (above 60 on the scale). Also similar to the Israeli sample, the Palestinian sample was generally well educated, with 66.1% reporting that they had completed college. As with the Israeli participants, Palestinians completed an omnibus survey of their political and social attitudes (see supplementary materials for full survey).

**Table 3:** Descriptive statistics and variable intercorrelations for Study 2.

	1	2	3	4	5	6	7	8	9	10	11
1. Perceived Group Power	-										
2. Social Dominance Orientation	-.02	-									
3. Conservatism	-.23***	.07	-								
4. Blatant Dehumanization	-.27***	-.09	.28***	-							
5. Trust	.03	.12*	-.16**	-.27***	-						
6. Hope	-.03	.18**	-.26***	-.32***	.65***	-					
7. Group-Based Guilt	-.02	.28***	-.21***	-.22***	.30***	.34***	-				
8. Hostile Emotions	-.21***	-.21***	.38***	.33***	-.40***	-.49***	-.38***	-			
9. Parochial Empathy	-.08	-.24***	.33***	.31***	-.44***	-.49***	-.47***	.54***	-		
10. Willingness to Negotiate	-.01	.04	-.13*	-.25	.40***	.43***	.04	-.27***	-.13*	-	
11. Willingness to Sacrifice Israeli Lives	-.15**	-.03	.10	.12*	-.10	-.12*	-.11*	.09	.19***	-.18**	-
*M*	60.68	30.62	71.41	37.03	31.61	23.86	15.00	70.60	73.94	45.37	46.76
*SD*	34.87	17.70	26.80	51.58	26.87	25.91	21.87	27.00	35.28	37.15	37.02
Quartiles	35.00, 69.00, 95.25	17.22, 31.50, 45.34	52.00, 76.00, 97.00	0.00, 35.50, 87.75	5.00, 28.50, 50.00	0, 15.00, 44.50	0, 3.00, 23.00	51.67, 70.00, 99.33	58.25, 93.50, 100.00	1.00, 48.50, 77.75	7.25, 47.00, 86.75

*Note*. Higher scores on perceived group power reflect a perception of Israel (i.e,. the outgroup) as more advantaged

*** p < .001

** p < .01

* p < .05

#### Measures

Although several of the constructs assessed in the Palestinian sample were identical to those assessed in the Israeli sample, there were some differences for other variables, particularly for some of the outcome measures. Due to the conflict’s asymmetry, direct parity was not possible for some items. For example, Palestinians do not have the military capacity to harm 1000 Israeli civilians, and we therefore adapted the item on acceptance of civilian casualties to capture similar underlying psychological processes (the tradeoff between ingroup and outgroup lives).

#### Age and gender

Participants indicated their age and gender (1 = male; 2 = female).

#### Education

Participants indicated whether they had: 1 = Completed primary education; 2 = Completed secondary education; 3 = Completed some college education; 4 = Completed College; 5 = Completed graduate school.

#### Social dominance orientation

The dominance sub-dimension of SDO was assessed using the same eight items as in Study 1, here on unmarked sliders anchored at 0 (‘Very much disagree’) and 100 (‘Very much agree’) (α = .62).

#### Conservatism

Participants were asked to indicate their political ideology by characterizing their levels of political, religious, and social conservatism on unmarked sliders anchored at 0 (‘Liberal’) and 100 (‘Conservative’) (α = .91).

#### Perceived group power

We assessed subjective perceptions of group power as in Study 1, here on unmarked sliders anchored at 0 (‘Definitely Palestinians’) and 100 (‘Definitely Israelis’).

#### Blatant dehumanization

Blatant dehumanization was assessed with the Ascent measure, as in Study 1. Here, participants were presented with the following target groups: Americans, Europeans, East Asians, Palestinians, Israelis, Israeli settlers, and Israeli peace activists. As with Study 1, we computed dehumanization by subtracting ratings of Israeli humanity from ratings of Palestinian humanity. For mean dehumanization responses across all target groups, see S2 Table.

#### Trust

We reasoned that individuals who dehumanized the outgroup would also be less likely to trust them, a key element promoting the likelihood of intergroup reconciliation [39]. We assessed trust of Israelis using three items: “I do not believe in the peaceful intentions of Israelis” (reverse-scored), “I trust that Israelis want to find a solution that will bring peace between Palestinians and Israelis”, and “If Israelis signed a political agreement, I trust that they would honor that agreement” [39]. Responses were provided on unmarked sliders anchored at 0 (‘Completely disagree’) and 100 (‘Completely agree’). The first item was not correlated with the remaining two items, from which we thus computed our composite measure (*r* = .33, *p* < .001).

#### Group-based guilt

We assessed group-based guilt as in Study 1, here on unmarked sliders anchored at 0 (‘Not at all’) and 100 (‘Very much so’) (*r* = .45, *p* < .001).

#### Emotional hostility

Hostile emotions were assessed as in Study 1, here on unmarked sliders anchored at 0 (‘Not at all’) and 100 (‘Very much so’) (α = .77).

#### Hope

We assessed hope using three of the four items assessed in Study 1: “I have hope regarding the peaceful resolution of the Israeli-Palestinian conflict”, “Under certain circumstances, and if all the core issues of the conflict are addressed, the Israeli-Palestinian conflict can be resolved”, and “I don’t ever expect to reach peace with the Israelis” (reverse-coded). Responses were provided on unmarked sliders anchored at 0 (‘Completely Disagree’) and 100 (‘Completely Agree’). The third item was weakly correlated with the other two items, so we computed our hope composite using the first two items (*r* = .50, *p* < .001).

#### Parochial empathy

Withholding empathy from the outgroup and reserving it for the ingroup is an important contributor to cycles of intergroup conflict [40]. Blatant dehumanization has previously been shown to negatively predict empathic responses to outgroup suffering [10]. We assessed parochial empathy [41] by asking participants: “How much compassion do you feel for Palestinians who have suffered as a result of the conflict”, and “How much compassion do you feel for Israelis who have suffered as a result of the conflict”. Each of these items was assessed on unmarked sliders anchored at 0 (‘None at all’) and 100 (‘A lot’), and parochial empathy was computed as the difference in expressed compassion towards Palestinian versus Israeli suffering.

#### Willingness to negotiate

We asked Palestinians to report their agreement with the following item: “How willing would you be for the Palestinians to enter direct negotiations with Israel in order to reach a final settlement?” Participants responded on an unmarked slider anchored at 0 (‘Not at all’) and 100 (‘Very much so’).

#### Willingness to sacrifice Israeli lives

Finally, we gave participants a version of the classic trolley dilemma [42] in order to examine how morally permissible they thought it was to prioritize saving one Palestinian life over 4 Israeli child civilians. Subsequently, participants were asked: “How morally permissible is it to save the 4 Israeli children and let the 1 Palestinian man die?” Responses were provided on an unmarked slider anchored at 0 (‘Absolutely the wrong thing to do’) and 100 (‘Absolutely the correct thing to do’), and then reverse scored such that higher scores reflect greater moral acceptance of sacrificing Israeli lives.

### Results

Descriptive statistics and variable inter-correlations can be found in Table 3.

As expected, Palestinians perceived their own group as significantly lower in power relative to Israelis (*M* = 60.68, *SD* = 34.86; one-sample t-test relative to the scale midpoint: *t*(354) = 5.77, *p* < .001; see Table 3). Thus, our sample of Palestinian participants shared the Israelis’ view that Palestinians were the low-powered party in an asymmetric relationship with Israel.

Consistent with Study 1, Palestinian blatant dehumanization of Israelis (versus Palestinians) was strong, with Israelis rated an average of 37.03 points lower than Palestinians on the Ascent scale. Moreover, blatant dehumanization of Israelis was also normative in our sample: 50% of participants rated Israelis over 35 points lower than Palestinians on the measure, and 25% of the sample rated Israelis nearly 88 points lower (i.e., almost at ceiling; see Table 3). When we examined absolute (rather than relative) ratings of Israelis on the Ascent scale, we observed that our sample of Palestinians rated Israelis similar to how Israelis rated Palestinians: closer in ‘evolvedness’ to the quadrupedal primate than to the ‘fully evolved’ human on the Ascent diagram (*M* = 45.03, *SD* = 39.25), *t*(353) = 2.38, *p* = .02.

Also similar to Study 1, we observed strikingly high levels of negativity and hostility: participants indicated low trust and hope, reported experiencing low group-based guilt, expressed high levels of emotional hostility towards Israelis, and exhibited high levels of parochial empathy. There was moderate support for negotiations, and a similarly moderate level of willingness to sacrifice one Palestinian life to save four Israeli children.

We next examined the extent to which dehumanization was associated with hostile intergroup attitudes and policy support. Following the same strategy as in Study 1, we performed multiple regression analyses for each of the outcome measures using SDO, conservatism and dehumanization as predictors, and controlling for perceived group power, age, gender and education (see Table 4). Similar to Study 1, more conservative participants were less trusting, less hopeful, less guilty, more emotionally hostile, expressed more parochial empathy, and were less willing to negotiate. Consistent with prior work on SDO among disadvantaged groups [43], [44], *lower* SDO (i.e., a greater desire for intergroup equality) was significantly associated with more rejectionist attitudes with respect to the advantaged group: less hope and guilt, more emotional hostility and parochial empathy. SDO was unassociated with willingness to negotiate, and neither of SDO or conservatism was associated with willingness to sacrifice Israeli lives.

**Table 4:** Simultaneous regressions predicting outgroup attitudes as a function of political ideology and dehumanization in Study 2.

	Trust R^2^ = .10		Hope R^2^ = .21		Group-Based Guilt R^2^ = .13		Hostile Emotions R^2^ = .27		Parochial Compassion R^2^ = .22		Willingness to Negotiate R^2^ = .11		Willingness to Sacrifice Israeli Lives R^2^ = .10	
	β	95% CI	Β	95% CI	β	95% CI	β	95% CI	β	95% CI	β	95% CI	β	95% CI
Perceived Group Power	-.05	-.17, .06	-.17**	-.27, -.07	-.11†	-.20, .00	-.07	-.18, .03	.02	-.08, .12	-.13*	-.24, -.03	-.11*	-.22, -.00
SDO	.09†	-.02, .20	.17**	.07, .28	.25***	.14, .33	-.23***	-.34, -.14	-.22***	-.32, -.12	.06	-.04, .17	-.02	-.13, .09
Conservatism	-.13*	-.24 -.02	-.25***	-.36, -.15	-.19**	-.28, -.08	.32***	.23, .44	.29***	.19, .39	-.13*	-.24, -.02	.06	-.05, .18
Age	-.02	-.13, .08	.03	-.07, .13	.02	-.08, .11	.01	-.09, .10	-.04	-.13, .06	.06	-.05, .16	-.03	-.14, .07
Gender	-.02	-.13, .09	-.02	-.12, .08	-.08	-.18, .02	.06	-.04, .15	.05	-.04, .15	.05	-.06, .15	.25***	.15, .36
Education	-.07	-.18, .04	-.11*	-.21, -.01	-.05	-.14, .05	.02	-.08, .12	.05	-.05, .14	.06	-.05, .16	-.06	-.17, .05
Blatant Dehumanization	-.23***	-.34, -.12	-.28***	-.38, -.17	-.13*	-.23, -.02	.20***	.10, .31	.21***	.11, .32	-.27***	-.38, -.16	.06	-.05, .17

*Note*. Higher scores on perceived group power reflect a perception of Israel (i.e,. the outgroup) as more advantaged.

*** *p* < .001

** *p* < .01

* *p* < .05

† *p* < .10

Most importantly for the current work, after controlling for power perceptions, ideology and demographics, blatant dehumanization was significantly associated with all of the outcome measures, with the exception of willingness to sacrifice Israeli lives. Specifically, Palestinians expressing higher levels of blatant dehumanization exhibited significantly less trust and hope, less guilt, more emotional hostility towards Israelis, more parochial empathy, and less willingness to negotiate with Israel. Results for all outcome measures were consistent further controlling for attribution of secondary emotions to Israelis (i.e., controlling for a subtle form of dehumanization).

Finally, in a set of analyses complementary to Study 1, we examined the role of subjective perceptions of group power on the dehumanization of Israelis. We observed that the more Palestinians felt that they were disadvantaged relative to Israelis, the less they blatantly dehumanized Israelis (see Table 3). Said another way, the more powerful Palestinians felt, the more they blatantly dehumanized Israelis (consistent with the link between subjective power perceptions and levels of dehumanization of Palestinians among Israelis). As in Study 1, we used the PROCESS macro ([32], Model 1) to examine whether subjective perceptions of group power moderated the association between blatant dehumanization of Israelis and the outcome measures (controlling for all other variables). We observed no evidence of moderation when we examined trust of Israelis (*b* = -.04, *p* = .42, 95% CI [-.14, .06], hope (*b* = .01, *p* = .84, 95% CI [-.08, .10], emotional hostility (*b* = .06, *p* = .19, 95% CI [-.03, .15], or parochial empathy (*b* = -.00, *p* = .92, 95% CI [-.10, .09]). On the other hand, there were marginally significant interaction effects on willingness to negotiate (*b* = -.09, *p* = .09, 95% CI [-.18, .01]) and willingness to sacrifice Israeli lives (*b* = .09, *p* = .07, 95% CI [-.01, .19]), and a significant interaction on group-based guilt (*b* = -.11, *p* = .02, 95% CI [-.20, -.01]. In all cases, the association between the outcome measure and blatant dehumanization was stronger the more *powerless* Palestinians perceived their group to be relative to Israelis.

## Comparison between Israeli and Palestinian samples

The data from Studies 1 and 2 suggest that both Palestinians and Israelis perceive that the two sides are involved in asymmetric conflict, and both agree that Israel as the more powerful party. Even more importantly for our purposes, these data suggest that blatant dehumanization, assessed in the context of ongoing conflict, is strong and consequential for both groups. In a final analysis, we sought to formally assess whether blatant dehumanization was of similar magnitude and consequentiality among our samples of these two groups.

We note that our samples of Israeli and Palestinian participants were not probability samples of the populations in each country, and thus the results cannot readily be generalized to the populations at large. Nevertheless, the samples we obtained were comparable on a number of levels. For example, each sample was balanced by gender (Israelis: 51.2% male; Palestinians: 55.8% male), included similar education levels (Israelis: 67.3% completed college; Palestinians: 66.1% completed college) and had similar political leanings (Israelis: 57.6% reported being at least slightly conservative (‘slightly’, ‘moderately’, or ‘strongly’ conservative); Palestinians: 61.7% at least slightly conservative (above 60 on a continuous 100-point scale)). The Israeli sample was approximately 10 years older, on average (Israelis: *M* age = 39.65, *SD* = 12.74; Palestinians: *M* = 27.11, *SD* = 8.90), consistent with the difference in median ages between these populations [45]. Given the overall similarity in the demographic features of our two samples, we thought it reasonable to compare across them, while including age, gender, education and conservatism as covariates in the analyses to account for any differences. Nevertheless, the caveat that the sample populations were not perfectly symmetrical should be kept in mind.

We first assessed whether the two samples differed in their subjective perceptions of overall group power with an analysis of covariance (ANCOVA), controlling for conservatism, SDO, and demographic variables (age, gender, education). We found that both Palestinians and Israelis perceived Israel to be the more powerful side, but that Israelis perceived the power disparity to be significantly greater, *F* (1, 830) = 66.17, *p* < .001, partial η^2^ = .07; we thus included perceived power as a covariate in all subsequent analyses.

We next assessed overall levels of dehumanization across the two samples using an ANCOVA, controlling for age, gender, education, conservatism, SDO, and subjective power perceptions. We observed a significant but small difference in outgroup dehumanization between Israelis and Palestinians, with Israelis showing slightly higher levels, *F*(1, 827) = 5.89, *p* = .02, η^2^ = .007. Next, we examined the difference across samples in outcome measures (again including the same set of control variables). Although some of the outcome measures we examined were unique across contexts, four of the outcome measures were either identical (i.e., group-based guilt; emotional hostility; the two hope items identical across samples) or very similar in content (i.e., willingness to negotiate). For these outcome measures, we examined how endorsement differed as a function of group membership: we found that Palestinians (versus Israelis) were more emotionally hostile (*F*(1, 828) = 14.77, *p* < .001, partial η^2^ = .02) and less hopeful (*F*(1, 829) = 43.50, *p* < .001, η^2^ = .05), and yet were significantly more willing to negotiate (*F*(1, 827) = 19.80, *p* < .001, η^2^ = .02). Palestinians and Israelis showed similar (low) levels of group-based guilt (*F* < 1). Results were consistent when the covariates were not included, with the exception that the difference in blatant dehumanization between groups was nonsignificant, *F* < 1.

Finally, we considered whether outgroup dehumanization *functioned* similarly for Israelis and Palestinians–i.e., whether the predictive validity of blatant dehumanization was similar for both groups. We first examined the relationship between blatant dehumanization and each of the four outcome measures shared across both Israeli and Palestinian surveys using the PROCESS macro ([32]; Model 1). We included age, sex, education, conservatism, SDO, and subjective power perceptions as covariates, and we controlled for the interaction terms between each of these variables and group membership (thus, our analyses tested the significance of the difference in the strength of the relationships between dehumanization and the outcome variables reported in Tables 2 and 4). These results revealed that dehumanization was similarly associated with the four outcomes that were measured similarly across both groups: for group-based guilt, emotional hostility, and hope, the interaction term between dehumanization and group membership did not significantly predict outcomes (group-based guilt: *b* = -.02, *p* = .74, 95% CI: [-.15, .11]; emotional hostility: *b* = -.10, *p* = .11, 95% CI: [-.23, .02]; hope: *b* = -.11, *p* = .11, 95% CI: [-.24, .03]. There was a marginally significant interaction for willingness to negotiate (*b* = -.13, *p* = .052, 95% CI: [-.27, .00]), suggesting that the association between blatant dehumanization and willingness to negotiate was somewhat stronger for Palestinians than for Israelis (although it was significant for both groups).

When we conducted these same interaction analyses without the covariates included, we obtained the same conclusions, with two exceptions: There was a significant interaction between group membership and blatant dehumanization predicting hostile emotions, such that the effects of blatant dehumanization were slightly stronger among Israelis compared to Palestinians (though significant across both groups): *b* = -.14, *p* = .03, 95% CI [-.26, -.01], and the interaction between group membership and blatant dehumanization on willingness to negotiate was non-significant (*b* = .07, *p* = .26, 95% CI [-.06, .21].

## Discussion

In sum, despite their disadvantaged status, Palestinians reported strikingly high levels of blatant dehumanization of their more powerful Israeli neighbors. Indeed, we observed that the participants in our Palestinian and Israeli samples dramatically dehumanized the outgroup, rating them as closer to animals than full humans. Not only was the *degree* of outgroup dehumanization quite high among the advantaged and disadvantaged group, but it was also quite potent: as with Israelis, Palestinians’ dehumanization of the outgroup was reliably and uniquely associated with aggressive attitudes and support for aggressive policies likely to feed cycles of intergroup violence.

Interestingly, although Palestinians feeling subjectively more powerless were somewhat less likely to blatantly dehumanize Israelis, dehumanization of Israelis was just as strongly (or more strongly) associated with the outcome variables among this group compared to Palestinians who felt more powerful.

## General discussion

Israeli and Palestinian participants surveyed during the 2014 Gaza war displayed extreme hostility towards outgroup members, even outgroup civilians explicitly uninvolved in hostilities: both groups reported highly negative outgroup-directed emotions, low concern for the other’s suffering, low levels of hope, and high support for collective aggression and endorsement of outgroup civilian casualties. These ratings were matched, among both groups, by strikingly high levels of blatant dehumanization: our samples of Palestinians and Israelis rated the other group nearly 40 points lower in ‘evolvedness’ than the ingroup, rating the outgroup to be closer to animals than full humans. Our results thus reflect both the dire realities of the Palestinian/Israeli conflict, as well as the unsavory but important fact that blatantly dehumanizing entire groups of people remains a feature of contemporary society [9], [46].

Beyond simply identifying high levels of intergroup hostility and dehumanization, our work makes several important theoretical contributions. For one, we extend prior empirical work on explicit blatant dehumanization conducted among advantaged group members [10] by examining this construct among members of a disadvantaged group. Moreover, we extend prior theorizing by specifically exploring how group power is associated with blatant outgroup dehumanization in a context marked by regular conflict. Here, we explored the role of power on blatant dehumanization in two ways: examining dehumanization as a function of membership in an advantaged versus disadvantaged group, and as a function of variation in subjective ratings of power among members of each group.

Our findings highlight a nuanced pattern of associations. On the one hand, we observed—among both Israelis and Palestinians—that the more individuals subjectively perceived that their group had power, the more likely they were to express blatant dehumanization of the outgroup (*r* = .14 among Israelis; *r* = .27 among Palestinians). In this way, our findings are consistent with earlier work that has suggested an association between feelings of power and dehumanization [23]. At the same time, the associations between subjective power and dehumanization were relatively weak. Moreover, feeling subjectively less powerful did not tend to affect the predictive utility of dehumanization: We found little evidence (among either group) that subjective power perceptions moderated the association between blatant dehumanization and our outcome measures (and indeed the only suggestive patterns indicated that blatant dehumanization had *more* predictive utility among Palestinians who felt more powerless).

Indeed, our analyses examining group membership are consistent with the idea that (at least in certain contexts) lacking power is not a decisive factor in determining the likelihood of blatant outgroup dehumanization. Although both Palestinians and Israelis in our study subjectively perceived Israel as the powerful group and Palestinians as the disempowered group, average levels of outgroup dehumanization across both samples were very similar (and very high). Furthermore, group membership did not significantly moderate the unique association between blatant dehumanization and intergroup hostility. Thus, blatant dehumanization was similarly associated among both groups with aggressive outcome variables (such as supporting collective violence), controlling for demographics, subjective power perceptions, and variables reflecting political ideology (i.e., SDO and conservatism).

Despite the contributions of our work, much remains to be understood. For one, although we document symmetric patterns of blatant dehumanization among Israelis and Palestinians, the reasons underlying this symmetry remain unclear. We think that one primary reason for the patterns that we observed here is that blatant dehumanization in part reflects overt perceptions of savagery, brutality, and cold-heartedness ([10], Study 5; [16]). In highly asymmetric violent conflicts, perceptions of savagery and barbarism may be a great equalizer: even if the advantaged group is able to lay greater claim to features associated with full humanity such as ‘sophistication’ and ‘advancement’, they may still be heavily (and equally) dehumanized by the disadvantaged group due to the perceived brutality of their actions. Whereas research on dehumanization of advantaged groups by disadvantaged groups has begun examining moderators such as desires for assimilation [47] and power primes [48], we suggest that perceived brutality also deserves further attention.

Although the primary purpose of this research was to examine blatant dehumanization among members of a minority/disempowered group, we also compared the prevalence and potency of blatant dehumanization across Israeli and Palestinian samples. For any cross-cultural comparison study, it is important to acknowledge the strengths and limitations of the samples collected. A benefit of the samples presented here is that they were not limited to a specific demographic (e.g., educated, liberals, men) [49]. Rather, our samples were relatively large community samples that included approximately equal numbers of men and women; each sample leaned slightly conservative, and represented a range of education levels, with approximately one third from each sample lacking a college degree. The size and heterogeneity of the samples gives us confidence that the results reported reflect the views of a wide range of the Israeli and Palestinian populations.

Since the samples were comparable to each other, we opted to compare them directly in a secondary analysis. However, the limitations of this analysis should be noted. First, there were some minor differences between the surveys. For example, the reference groups included in the dehumanization measure were different across samples, and the ideological covariates (i.e., SDO, conservatism) and outcome measures (willingness to negotiate) were assessed with a 7-point Likert scale for one group, and a continuous slider for the other (however, note that all measures were z-scored to account for scale differences). Moreover, willingness to sacrifice outgroup lives was assessed differently across groups, and willingness to negotiate was assessed in Israelis towards the two main Palestinian political groups (‘Mahmoud Abbas’, the Palestinian president who leads the Fatah political party, and ‘Hamas’, the other political party that controls Gaza), whereas this item was assessed in Palestinians towards only one group (‘Israelis’). Although this approach maintained ecological validity, it introduced slight differences into the measures across samples. However, there is reason to believe that these small methodological differences had little effect on the main conclusions. Specifically, our previous research has shown dehumanization judgments to be stable in American and Hungarian participants, regardless of the other target groups included on the scale [10], or the type of scale used (i.e., continuous slider versus Likert scale). It also seems unlikely that the slight differences in our measurement of willingness to negotiate dramatically impacted the main conclusions, particularly given that the results for this outcome measure were similar to the results for outcomes that were worded identically across groups. Nevertheless, future work comparing across samples could certainly opt to maximize similarity of the surveys, rather than ecological validity.

Second, although relatively large and diverse, the samples we collected were not national probability samples, and thus they cannot be considered fully representative of the underlying populations. Moreover, the mean age of the samples differed: the Israeli sample was approximately 10 years older than the Palestinian sample (however, note that this reflects the actual difference in mean age between these populations). One way to mitigate this concern is to account for the effects of age statistically, using them as covariates in the analyses. Here, we controlled for age, gender, and education (as well as political conservatism) in all regression analyses.

Finally, data from Palestinian sample was collected right as the war in Gaza was ending, approximately 2 weeks after the Israeli sample was gathered. Although we think it unlikely that negative perceptions would change markedly over this time, it is important to acknowledge this difference.

To further increase confidence in our conclusions and examine their generalizability, future work should re-examine blatant dehumanization among Israelis and Palestinians using representative samples gathered simultaneously, and extend this work to a range of asymmetric contexts, including those that do not involve violent conflict and war. Indeed, the conflictual setting we examined is associated with perceptions of illegitimacy and competition over victimhood status [50], both processes that might be expected to accentuate outgroup dehumanization. In social contexts where the hierarchy is relatively stable and imbued with greater legitimacy (e.g., the longstanding caste system in India), it is possible that disadvantaged groups (e.g., India’s Dalit, or ‘untouchables’) refrain from blatantly dehumanizing those at the top, and perhaps even internalize their own dehumanized status. Finally, it is worth noting that we conducted our study in the midst of a particularly ‘hot’ period (i.e., wartime) of a reliably simmering conflict. Although examining blatant dehumanization amidst an active and consequential intergroup conflict has important advantages, it would be interesting to compare our results to times of relative peace (or ‘colder’ conflict) to concretely isolate the role of active warfare.

Overall, this work adds substantial weight to the recent evidence for the importance of blatant outgroup dehumanization, showing that it can take root not only among those groups occupying the upper echelons of power and status but also among those at the bottom. Our findings argue for the importance of continued research in this area. If, as UNESCO claims, “wars begin in the minds of men [and women]”, it is critical that we understand how and why individuals come to openly perceive their adversaries as animals unworthy of moral consideration, so we can assess efforts employed to erode this imposing psychological edifice.

## Supporting information

S1 Table Mean blatant dehumanization among Israelis in Study 1, assessed using the Ascent measure. (DOCX)

S2 Table Mean blatant dehumanization among Palestinians in Study 2, assessed using the Ascent measure. (DOCX)

S1 File Full surveys. (DOCX)
