# Supplementary material for: The enemy as animal: Symmetric dehumanization during asymmetric warfare
Source: PLoS One. 2017 Jul 26;12(7):e0181422. doi: 10.1371/journal.pone.0181422 (PMC5528981; doi:10.1371/journal.pone.0181422)
Supplement: S1 Table — (DOCX) [file pone.0181422.s001.docx]

**S1 Table. Mean blatant dehumanization among Israelis in Study 1, assessed using the Ascent measure.**

| Target | Mean *(SD)* | Quartiles  (25, 50, 75) |
| --- | --- | --- |
| Israelis | 81.01 (18.96) | 70, 85, 99 |
| Americans | 74.06 (22.04)*** | 58, 80, 92 |
| Europeans  Arab Israelis  Arabs  Muslims  Gazans | 74.37 (23.07)***  55.91 (28.30)***  47.38 (30.53)***  44.00 (31.34)***  43.63 (31.18)*** | 60, 80, 94  36, 53, 80  22, 50, 72  17, 41 68  16, 41, 69 |
| Palestinians | 41.21 (32.46)*** | 10, 35, 70 |
| Hamas Members | 21.34 (30.46)*** | 0, 3, 40 |
|  |  |  |

*Note.* Asterisks reflect significant difference in average Ascent rating from ingroup (Israelis). * *p* < .05 ** *p* < .01 *** *p* < .001
