# Supplementary material for: The enemy as animal: Symmetric dehumanization during asymmetric warfare
Source: PLoS One. 2017 Jul 26;12(7):e0181422. doi: 10.1371/journal.pone.0181422 (PMC5528981; doi:10.1371/journal.pone.0181422)
Supplement: S2 Table — (DOCX) [file pone.0181422.s002.docx]

**S2 Table. Mean blatant dehumanization among Palestinians in Study 2, assessed using the Ascent measure.**

| Target | Mean *(SD)* | Quartiles  (25, 50, 75) |
| --- | --- | --- |
| Palestinians | 81.74 (25.99) | 69, 94, 100 |
| Europeans | 76.11 (26.00)** | 66, 85, 99 |
| East Asians | 69.45 (27.91)*** | 51, 73, 95 |
| Americans | 67.91 (32.32)*** | 51, 78, 97 |
| Israeli Peace Activists | 57.86 (33.95)*** | 30, 64, 92 |
| Israelis  Israeli Settlers | 45.03 (39.25)***  36.04 (39.03)*** | 3, 39, 86  0, 16, 73 |

*Note.* Asterisks reflect significant difference in average Ascent rating from ingroup (Israelis). * *p* < .05 ** *p* < .01 *** *p* < .001
