# Supplementary material for: The enemy as animal: Symmetric dehumanization during asymmetric warfare
Source: PLoS One. 2017 Jul 26;12(7):e0181422. doi: 10.1371/journal.pone.0181422 (PMC5528981; doi:10.1371/journal.pone.0181422)
Supplement: S1 File — (DOCX) [file pone.0181422.s003.docx]

**Full Surveys**

**Israeli Survey**

Imagine that the pictures on the next pages are depictions of different kinds of social groups. Groups in society can be organized in different ways. Each circle in the picture below represents one individual. For each picture, please tell us how much you like groups like this.

How much do you like groups like this?

**[1= strongly dislike, 2= moderately dislike, 3= slightly dislike; 4= neutral; 5=slightly like; 6= moderately like; 7= strongly like]**

**a.**

**
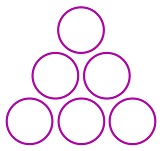
**

b.


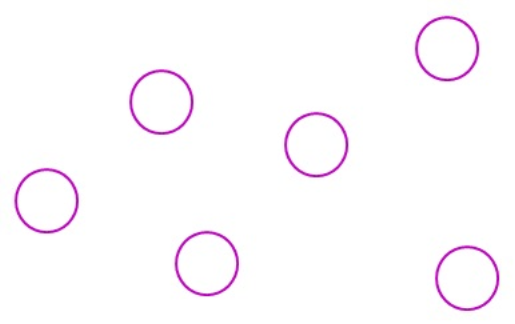


**c.**

**
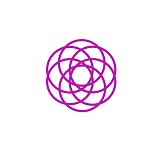
**

**d.**


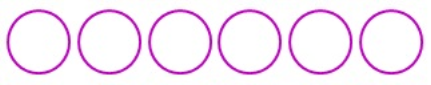


**e.**


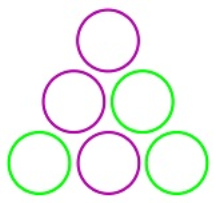


**f.**


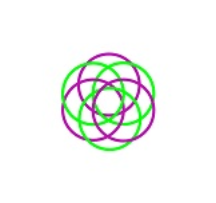


**g.**

**
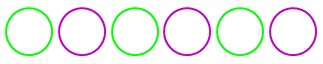
**

**h.**


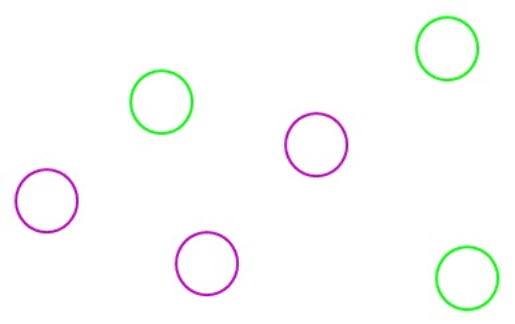


**i.**

**
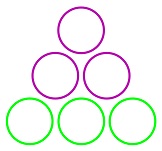
**

**j.**


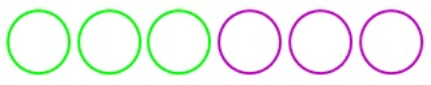


**k.**

**
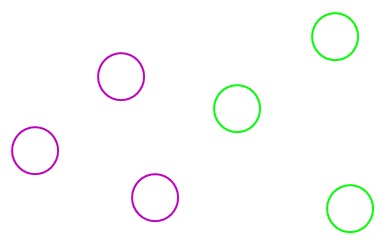
**

**Ascent Dehumanization**

Some people believe that people can vary in how human-like they seem. According to this view, some people seem highly evolved whereas others seem no different than lower animals. Using the image below as a guide, indicate using the sliders how evolved you consider the average member of each group to be.  [**0= left side of the image below; 100= right side of the image below]**


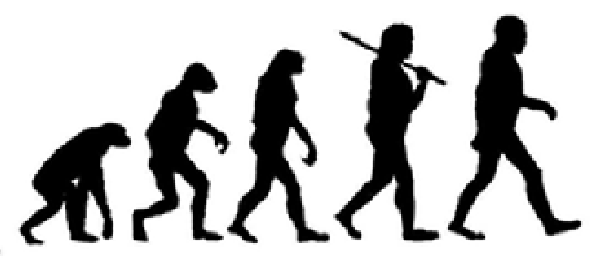


1. Europeans
2. Palestinians
3. Arabs
4. Muslims
5. Israelis
6. Residents of Gaza
7. Members of Hamas
8. Americans
9. Arab-Israelis

**Infrahumanization**

To what extent are Palestinians *capable* of experiencing each of the following emotions: [1=Not capable at all; 7= very capable]

1. Shame
2. Hope
3. Love
4. Guilt
5. Bitterness
6. Compassion
7. Passion
8. Remorse

**Meta-Ascent Dehumanization**

We are interested in your perception about what *Palestinians* think of Israelis. How do you think **Palestinians** would rate Israelis on the scale below?


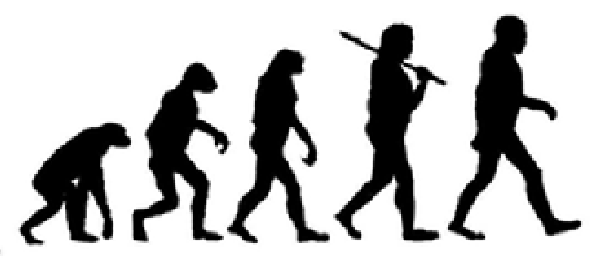


**Hope [1=Strongly Disagree ; 7= Strongly Agree[**

1. I have hope regarding the peaceful resolution of the Israeli-Palestinian conflict
2. Israel should stop trying to resolve the conflict, because it is impossible
3. I don’t expect to ever achieve peace with the Palestinians
4. Under certain circumstances, and if all the core issues of the conflict are addressed, the Israeli Palestinian conflict can be resolved.

**Emotions [1=Not at all; 7 = Very much so]**

Guilt regarding Israel's treatment of the Palestinians

Shame regarding Israel's behaviour towards the Palestinians

Hatred towards the Palestinians

Anger regarding Palestinians' actions towards Israel

Hostility towards the Palestinians

Empathy towards the Palestinians

Fear from actions the Palestinians may carry out

**Perceived Power [1=Definitely Palestinians; 7= Definitely Israel]**

1. In general, when you think about power relations between Israel and the Palestinians, which side do you think has more power?
2. In general, when you think about military power differences between Israel and the Palestinians, which side do you think has more power?
3. In general, when you think about legitimacy and support in the international community, which side do you think has the upper hand?
4. In general, when you think about economic power differences between Israel and the Palestinians, which side do you think has more power?
5. In general, when you think about the battle on social media for winning the world’s ‘hearts and minds’, which side do you think has more power?

With regards to the ongoing war in Gaza (“Operation Pillar of Defense”):

1. Which side is winning the war in a military sense?
2. Which side is winning the war in terms of sympathy around the world?
3. Which side is behaving more morally?

We would like you think about how the balance of power between Israelis and Palestinians might *change* in the coming 5-10 years.

1. Over the next 5-10 years, how will Israel’s position relative to the Palestinians in terms of overall power change : **[1=will become definitely weaker; 4= no change; 7= will become definitely stronger]**
2. Over the next 5-10 years, how will Israel’s position relative to the Palestinians in terms of military power change?
3. Over the next 5-10 years, how will Israel’s position relative to the Palestinians in terms of legitimacy and support in the international community power change?

**Social Dominance Orientation [1= Strongly Disagree; 7=Strongly Agree]**

1. Some groups of people must be kept in their place.
2. It’s probably a good thing that certain groups are at the top and other groups are at the bottom.
3. An ideal society requires some groups to be on top and others to be on the bottom.
4. Some groups of people are simply inferior to other groups.
5. Groups at the bottom are just as deserving as groups at the top.
6. No one group should dominate in society.
7. Groups at the bottom should not have to stay in their place.
8. Group dominance is a poor principle.
9. To what extent do you support a two-state solution as a resolution to the Israeli/Palestinian conflict?
10. To what extent do you support a one-state solution as a resolution to the Israeli/Palestinian conflict?

Consider the Israeli-Palestinian conflict. Several different solutions have been proposed for this conflict. Please indicate how favorable you would find each of the following solutions to be:

1. Dividing the land into a separate state for each group, with the Palestinian state including all of the West Bank and Gaza.
2. Dividing the land into a separate state for each group, with the Palestinian state including most of the West Bank and Gaza, with Palestinians given some land from the Negev in exchange for the settlements.
3. Maintaining things as they are (i.e., no agreement with the Palestinians).
4. Creating a single democratic state for all the people (Israelis and Palestinians) on all the land, in which each person receives one vote regardless of group membership.
5. Creating a bi-national state, in which the two groups would coexist as separate communities within a single state, with each group running their own affairs but participating equally in a single federal governmental structure.
6. Absorbing the West Bank and Gaza into Israel without giving Palestinians a right to vote.
7. Absorbing the West Bank and Gaza into Israel and forcing the Palestinians to go to Jordan.

**Willingness to negotiate [1=not at all; 7= Very much so]**

1. How willing would you be for Israel to negotiate with a Palestinian side led by Mahmoud Abbas?

2. How willing would you be for Israel to enter direct negotiations involving Hamas in order to reach a final settlement?

**Concession Making [1=not at all; 7= Very much so]**

1. In return for a full and final agreement and the end of the conflict, to what extent do you support Israel fully renouncing control for Gaza, the West Bank, and the Arab neighbourhoods in East Jerusalem?

2. To what extent do you support joint sovereignty over the holy sites in Jerusalem?

3. To what extent do you support a peace settlement that would include monetary compensation for Palestinian refugees and recognition of their right to return to Israel, but would not include actual right of return for refugees?

4. To what extent do you believe that united Jerusalem is ‘ours forever’ and should never be divided?

**Acceptance of Civilian Casualties**

Assuming the following scenario is taking place: An Israeli soldier is fighting with his combat unit in a neighborhood in Gaza. He is shot and wounded by a Palestinian militant. In order to save him, one of the other soldiers in the combat unit needs to fire a mortar shell back. However, this is a dense urban neighborhood that contains Palestinian civilians. What is the maximum number of Palestinian civilian casualties that you think is acceptable in order to save the soldier’s life **(please choose a number of Palestinian casualties beyond which you would no longer endorse the bombing; please choose a number between 0 and 1,000).**

**Collective Aggression [1= Strongly Disagree; 7=Strongly Agree]**

1. I think it is unacceptable to torture Palestinians even if it leads to useful information about Palestinian militants.
2. I support the continuation of the siege on Gaza.
3. I think that Israel should open border crossings to allow Palestinians in Gaza requiring medical attention outside to receive it.
4. As long as Hamas continues to fire rockets at Israel, I think it is justified to bomb Palestinian schools and hospitals.
5. Israel should allow humanitarian organization to enter the Gaza strip in order to aid its people
6. As long as there is no peace with the Palestinians, there is no reason for Israel to provide electricity and water to the Palestinians
7. To what extent do you support hurting Palestinians in order to "teach them a lesson"?
8. Israel should use live fire to disperse Palestinian protests, even at the cost of hurting civilians and bystanders.

1. Leftists should not be allowed to speak out against the military operation during these times.

2. Groups like the “Lions of the Shadow” and “El-Yahud” have a right to act violently against Leftists who hurt our nation’s unity.

**Political Conservatism**

How would you define your political orientation? **[1=Extreme Right; 7=Extreme Left]**

What political party did you vote for in the last election? _______

**Palestinian Survey**

What is your age?

What is you gender?

- Male
- Female

What is your highest education level?

- completed elementary school (1)
- completed secondary school (2)
- some college (3)
- finished undergraduate degree (4)
- finished graduate degree (5)

What is your primary language?

- Arabic (1)
- English (2)
- Other, please specify below (3) ____________________

With what group do you most strongly identify?

- Palestinian (1)
- Arab (2)
- Other, please specify below (3) ____________________

How strongly do you identify with that group? **[slider, anchored at ‘not at all’ and ‘very strongly’]**

With what religious group do you most strongly identify?

- Muslim (1)
- Christian (2)
- Other, please specify below (3) ____________________

How strongly do you identify with that religious group? **[slider, anchored at ‘not at all’ and ‘very strongly’]**

**Conservatism**

Characterize yourself politically [slider, anchored at ‘very liberal’ and ‘very conservative’]

Characterize yourself religiously [slider, anchored at ‘very liberal’ and ‘very conservative’]

Characterize yourself socially [slider, anchored at ‘very liberal’ and ‘very conservative’]

**Social Dominance Orientation  [0=Strongly Disagree; 100=Strongly Agree]**

Sometimes other groups must be kept in their place

It’s probably a good thing that certain groups are at the top and other groups are at the bottom

An ideal society requires some groups to be on top and others to be on the bottom

Some groups of people are simply inferior to other groups

Groups at the bottom are just as deserving as groups at the top

No one group should dominate in society

Groups at the bottom should not have to stay in their place

Group dominance is a poor principle

This question is to ensure you are paying attention. Please move the slider to the far left

**Prejudice**

Indicate below how cold (unfavorable) or warm (favorable) you feel towards each of the following groups

______ Palestinians

______ Europeans

______ East Asians

______ Americans

______ Israelis

______ Israeli Settlers

______ Israeli Peace Activists

**Ascent Dehumanization**

Some people believe that people can vary in how human-like they seem. According to this view, some people seem highly civilized whereas others seem no different than lower animals. Using the image below as a guide, indicate using the sliders how civilized you consider the average member of each group to be   .  [**0= left side of the image below; 100= right side of the image below]**

______ Palestinians

______ Europeans

______ East Asians

______ Americans

______ Israelis

______ Israeli Settlers

______ Israeli Peace Activists

**Infrahumanization - Israeli**

To what extent are Israelis capable of experiencing each of the following emotions .  [**0= Not capable at all; 100= Very Capable]**

______ Shame

______ Hope

______ Love

______ Guilt

______ Bitterness

______ Compassion

______ Passion

______ Remorse

**Infrahumanization - Palestinian**

To what extent are Palestinians capable of experiencing each of the following emotions

______ Shame

______ Hope

______ Love

______ Guilt

______ Bitterness

______ Compassion

______ Passion

______ Remorse

**Meta-Ascent Dehumanization**

We are interested in your perception about what Israelis think of Palestinians. How do you think Israelis would rate Palestinians on the scale below?

**Meta-Blatant Dehumanization**

Israelis perceive Palestinians to be sub-human

Israelis think of Palestinians as animal-like

Israelis think Palestinians are beasts

Israelis consider Palestinians to belong to a lower form of civilization

Israelis think of Palestinians as vermin

**Trust and Hope  [0=Completely Disagree; 100=Completely Agree]**

I do not believe in the peaceful intentions of the Israelis

I trust that Israelis want to find a solution that will bring peace between Palestinians and Israelis

If Israelis signed a political agreement, I trust that they would honor that agreement

I have hope regarding the peaceful resolution of the Israeli-Palestinian conflict

I don’t expect to ever achieve peace with the Israelis

Under certain circumstances, and if all the core issues of the conflict are addressed, the Israeli Palestinian conflict can be resolved

**Emotions [0=Not at all; 100=Very Much so]**

Please indicate how much you feel each of the following emotions

Hatred towards the Israelis

Fear from actions the Israelis may carry out

Anger regarding Israelis actions towards Palestine

Hostility towards the Israelis

Empathy towards the Israelis

Shame regarding Palestinians’ behaviour towards the Israelis

Guilt regarding Palestinian treatment of the Israelis

**Perceived Power [0= Definitely Israelis; 100= Definitely Palestinians]**

In general, when you think about power relations between Israel and the Palestinians, which side do you think has more power?

In general, when you think about legitimacy and support in the international community, which side do you think has the upper hand?

In general, when you think about economic power differences between Israel and the Palestinians, which side do you think has more power?

In general, when you think about the battle on social media for winning the World’s ‘hearts and minds’, which side do you think has more power?

With regards to the ongoing war in Gaza

Which side is winning the war in terms of sympathy around the world?

Which side is behaving more morally?

We would like you to think about how the balance of power between Israelis and Palestinians might change in the coming 5-10 years

In the next 5 to 10 years, how will the balance of power between Israelis and Palestinians change?

In the next 5 to 10 years, how will Israel’s military position change relative to the Palestinians?

In the next 5 to 10 years, how will the legitimacy of the Palestinian viewpoint and international support received change compared to that received by Israel?

**Negotiations**

How willing would you be for the Palestinians to enter direct negotiations with Israel in order to reach a final settlement? **[0-100 slider, anchored at ‘not at all’ and ‘very’]**

**Parochial Empathy**

How much compassion do you feel for Palestinians who have suffered as a result of the conflict? **[0-100 slider, anchored at ‘not at all’ and ‘very much’]**

How much compassion do you feel for Israelis who have suffered as a result of the conflict? **[0-100 slider, anchored at ‘not at all’ and ‘very much’]**

**Willingness to Sacrifice Israeli Lives**

Final Question: Please imagine the following scenario: on the steep road to Jericho, an Israeli settler stops his car by the side of the road and jumps out to talk to a friend across the road. Just then, a Palestinian man driving the same road loses control of his car when his tire explodes and hits the parked car, which has the Israeli settler&#39;s 4 young children in it. Both cars start slowly rolling towards the edge of a cliff; the Palestinian man is unconscious and the Israeli children are too young to do anything. You know that both cars will roll over the edge and kill everybody on board if nothing is done. You are the closest one to the accident and have just enough time to run up to one of the cars and step on the brake, but there is only enough time for you to do this with one of the cars -- the other car will definitely fall, killing whoever is inside.

How morally permissible is it to save the 4 Israeli children and let the 1 Palestinian man die? **[0-100 slider, anchored at ‘not at all’ and ‘completely’]**
